# Supplementary material for: The Associations of Erythrocyte Fatty Acids with Whole Blood Mineral Elements in Children
Source: Nutrients. 2022 Jan 30;14(3):618. doi: 10.3390/nu14030618 (PMC8838319; doi:10.3390/nu14030618)
Supplement: Supplementary file 1 [file nutrients-14-00618-s001.zip › nutrients-1565937-supplementary.pdf]

Supplement Table S1 Partial correlation analysis of mineral elements with erythrocyte FAs in children according to P50 stratification of whole blood Cu status.

FAs	Ca				Mg				Fe				Cu				Zn			
	Cu<P50		Cu≧P50		Cu<P50		Cu≧P50		Cu<P50		Cu≧P50		Cu<P50		Cu≧P50		Cu<P50		Cu≧P50	
	P^a	P_{FDR}^b	P^a	P_{FDR}^b	P^a	P_{FDR}^b	P^a	P_{FDR}^b	P^a	P_{FDR}^b	P^a	P_{FDR}^b	P^a	P_{FDR}^b	P^a	P_{FDR}^b	P^a	P_{FDR}^b	P^a	P_{FDR}^b
C16:0	0.610	0.986	0.298	0.560	0.427	0.952	0.001	**0.004**	0.297	0.675	0.279	0.941	0.169	0.410	0.684	0.958	0.253	0.253	0.756	0.873
C18:0	0.897	0.986	0.585	0.707	0.451	0.952	0.001	**0.001**	0.979	0.979	0.422	0.941	0.312	0.410	0.379	0.958	0.194	0.208	0.470	0.845
SFAs	0.917	0.986	0.531	0.707	0.364	0.952	0.001	**0.001**	0.603	0.886	0.393	0.941	0.174	0.410	0.418	0.958	0.191	0.208	0.815	0.873
**C18:1n-9**	0.396	0.986	0.974	0.974	0.365	0.952	0.008	**0.016**	0.937	0.979	0.206	0.941	0.076	0.410	0.952	0.958	0.007	**0.035**	0.644	0.845
**C24:1n-9**	0.453	0.986	0.156	0.333	0.054	0.814	0.001	**0.001**	0.367	0.688	0.822	0.941	0.094	0.410	0.711	0.958	0.003	**0.023**	0.223	0.835
**MUFAs**	0.369	0.986	0.547	0.707	0.160	0.892	0.002	**0.004**	0.671	0.886	0.510	0.941	0.077	0.410	0.897	0.958	0.003	**0.023**	0.876	0.876
C18:3n-3	0.491	0.986	0.532	0.707	0.178	0.892	0.363	0.777	0.650	0.886	0.278	0.941	0.383	0.410	0.603	0.958	0.135	0.169	0.462	0.845
C20:5n-3	0.961	0.986	0.028	0.104	0.775	0.953	0.198	0.371	0.298	0.675	0.633	0.941	0.344	0.410	0.272	0.958	0.053	0.088	0.359	0.845
C22:6n-3	0.351	0.986	0.007	0.080	0.681	0.953	0.014	**0.016**	0.094	0.675	0.673	0.941	0.261	0.410	0.937	0.958	0.080	0.109	0.572	0.845
n-3PUFAs	0.492	0.986	0.011	0.080	0.953	0.953	0.017	**0.025**	0.124	0.675	0.864	0.941	0.257	0.410	0.951	0.958	0.059	0.088	0.539	0.845
**C18:2n-6**	0.986	0.986	0.825	0.884	0.651	0.953	0.013	**0.016**	0.746	0.886	0.690	0.941	0.471	0.471	0.659	0.958	0.013	**0.048**	0.054	0.815
C20:4n-6	0.901	0.986	0.022	0.104	0.934	0.953	0.026	**0.033**	0.143	0.675	0.941	0.941	0.315	0.410	0.860	0.958	0.042	0.079	0.313	0.845
n-6PUFAs	0.955	0.986	0.144	0.333	0.886	0.953	0.016	**0.018**	0.315	0.675	0.855	0.941	0.367	0.410	0.946	0.958	0.022	**0.052**	0.151	0.835
PUFAs	0.897	0.986	0.107	0.322	0.903	0.953	0.015	**0.016**	0.283	0.675	0.887	0.941	0.350	0.410	0.958	0.958	0.024	**0.052**	0.176	0.835
Total FAs	0.854	0.986	0.613	0.707	0.508	0.952	0.001	**0.001**	0.768	0.886	0.534	0.941	0.150	0.410	0.662	0.958	0.024	**0.052**	0.676	0.845

a Adjustment: age and sex.

b The P-value was adjusted using false discovery rate method for multiple testing

Abbreviations: SFAs-saturated fatty acids; MUFAs- monounsaturated fatty acids; PUFAs-polyunsaturated fatty acids; P50-the 50th percentile.

Supplement Table S2 Partial correlation analysis of mineral elements with erythrocyte FAs in children according to P50 stratification of whole blood Zu status.

FAs	Ca				Mg（				Fe				Cu（				Zn			
	Zn<P50		Zn≧P50		Zn<P50		Zn≧P50		Zn<P50		Zn≧P50		Zn<P50		Zn≧P50		Zn<P50		Zn≧P50	
	P^a	P_{FDR}^b	P^a	P_{FDR}^b	P^a	P_{FDR}^b	P^a	P_{FDR}^b	P^a	P_{FDR}^b	P^a	P_{FDR}^b	P^a	P_{FDR}^b	P^a	P_{FDR}^b	P^a	P_{FDR}^b	P^a	P_{FDR}^b
C16:0	0.232	0.560	0.802	0.843	0.897	0.989	0.024	0.084	0.499	0.735	0.775	0.908	0.191	0.220	0.986	0.992	0.744	0.930	0.372	0.372
C18:0	0.336	0.560	0.590	0.843	0.361	0.989	0.059	0.098	0.946	1.000	0.431	0.908	0.485	0.485	0.551	0.992	0.930	0.930	0.335	0.372
SFAs	0.293	0.560	0.720	0.843	0.645	0.989	0.034	0.084	0.736	1.000	0.659	0.908	0.290	0.311	0.801	0.992	0.734	0.930	0.354	0.372
C18:1n-9	0.406	0.609	0.843	0.843	0.893	0.989	0.073	0.109	0.992	1.000	0.516	0.908	0.039	0.058	0.911	0.992	0.596	0.930	0.059	0.335
**C24:1n-9**	0.713	0.841	0.208	0.780	0.505	0.989	0.321	0.321	0.883	1.000	0.241	0.908	0.026	**0.049**	0.783	0.992	0.662	0.930	0.199	0.335
**MUFAs**	0.602	0.820	0.629	0.843	0.837	0.989	0.112	0.129	0.869	1.000	0.383	0.908	0.029	**0.049**	0.992	0.992	0.735	0.930	0.078	0.335
C18:3n-3	0.729	0.841	0.374	0.843	0.620	0.989	0.127	0.136	0.291	0.623	0.972	0.972	0.082	0.103	0.633	0.992	0.496	0.930	0.113	0.335
**C20:5n-3**	0.229	0.560	0.181	0.780	0.917	0.989	0.091	0.113	0.506	0.735	0.668	0.908	0.010	**0.025**	0.421	0.992	0.720	0.930	0.344	0.372
**C22:6n-3**	0.048	0.486	0.068	0.780	0.949	0.989	0.059	0.098	0.786	1.000	0.619	0.908	0.007	**0.024**	0.423	0.992	0.752	0.930	0.326	0.372
**n-3PUFAs**	0.065	0.486	0.114	0.780	0.989	0.989	0.039	0.084	0.684	0.735	0.654	0.908	0.005	**0.024**	0.432	0.992	0.829	0.930	0.291	0.372
C18:2n-6	0.846	0.907	0.806	0.843	0.810	0.989	0.011	0.084	0.649	0.735	0.779	0.908	0.049	0.067	0.884	0.992	0.447	0.930	0.139	0.335
**C20:4n-6**	0.132	0.560	0.269	0.806	0.950	0.989	0.082	0.111	0.707	0.884	0.569	0.908	0.003	**0.024**	0.378	0.992	0.926	0.930	0.201	0.335
**n-6PUFAs**	0.303	0.560	0.478	0.843	0.900	0.989	0.030	0.084	0.689	0.795	0.848	0.908	0.008	**0.024**	0.668	0.992	0.713	0.930	0.157	0.335
**PUFAs**	0.254	0.560	0.415	0.843	0.910	0.989	0.029	0.084	0.686	0.735	0.823	0.908	0.007	**0.024**	0.636	0.992	0.725	0.930	0.166	0.335
**Total FAs**	0.928	0.928	0.547	0.843	0.772	0.989	0.025	0.084	1.000	1.000	0.645	0.908	0.026	**0.049**	0.940	0.992	0.683	0.930	0.168	0.335

a Adjustment: age and sex.

b The *P*-value was adjusted using false discovery rate method for multiple testing

Abbreviations: SFAs-saturated fatty acids; MUFAs- monounsaturated fatty acids; PUFAs-polyunsaturated fatty acids; P50-the 50th percentile.
